# Supplementary figures and images for: MALDI-TOF mass spectrometry for the identification of freshwater snails from Senegal, including intermediate hosts of schistosomes
Source: PLoS Negl Trop Dis. 2021 Sep 13;15(9):e0009725. doi: 10.1371/journal.pntd.0009725 (PMC8489727; doi:10.1371/journal.pntd.0009725)

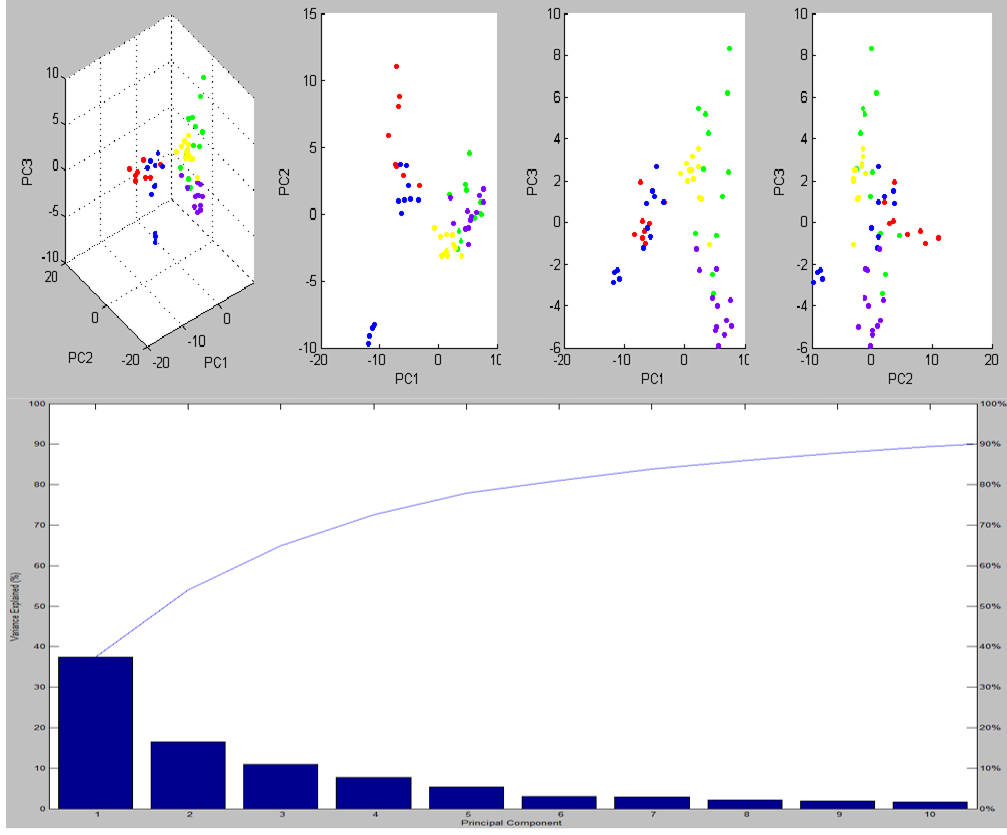

Supplement: S1 Fig — The figure shows the contribution of ten principal components to the profiling classification in plot of percentage explained variance of PC. Red dots: protocol H1, green dots: protocol H2, blue dots: protocol H3, yellow dots: FH and purple dots: F. (TIF) [file pntd.0009725.s001.tif]

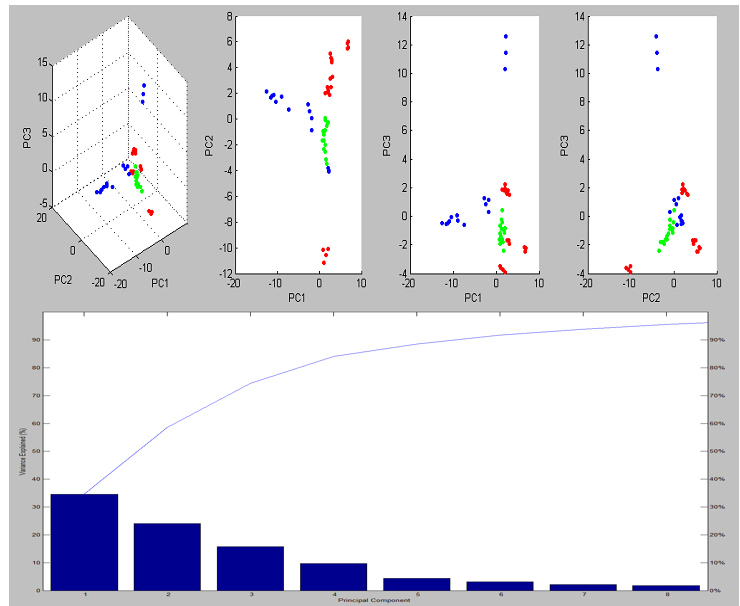

Supplement: S2 Fig — The figure shows the contribution of eight principal components to the profiling classification in plot of percentage explained variance of PC. Red dots: protocol F1, green dots: protocol F2, blue dots: protocol F3. (TIF) [file pntd.0009725.s002.tif]

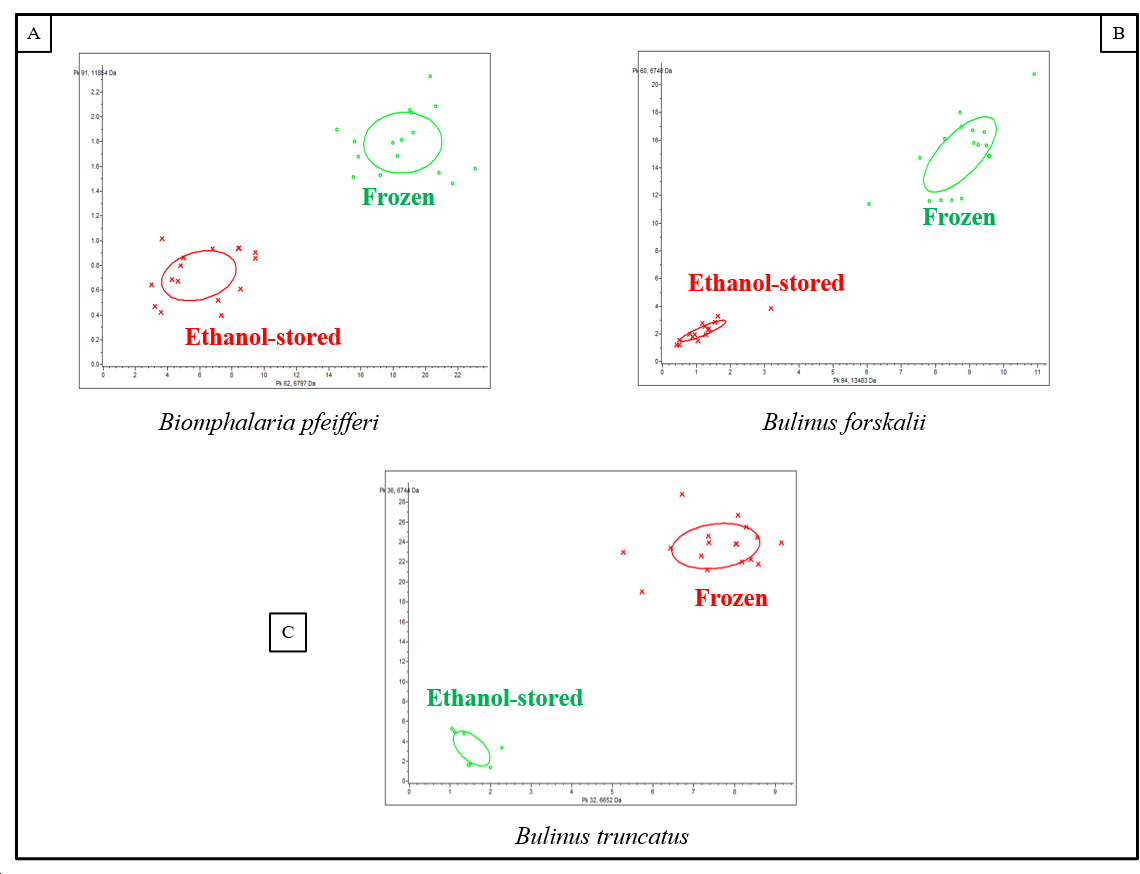

Supplement: S3 Fig — (A) Bi. pfeifferi, (B) Bu. forskalii, (C) Bu. truncatus. (TIF) [file pntd.0009725.s003.tif]

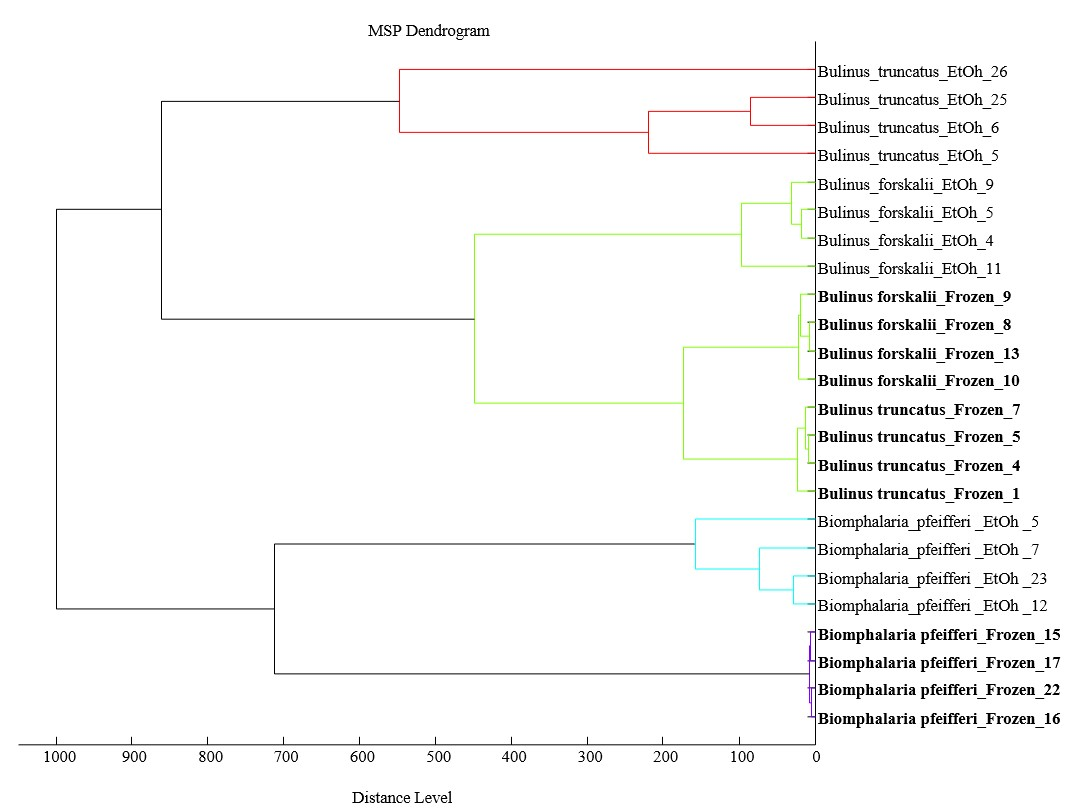

Supplement: S4 Fig — Frozen specimens are indicated in Bold and ethanol-stored specimens are not in Bold. (TIF) [file pntd.0009725.s004.tif]
