# Supplementary material for: MALDI-TOF mass spectrometry for the identification of freshwater snails from Senegal, including intermediate hosts of schistosomes
Source: PLoS Negl Trop Dis. 2021 Sep 13;15(9):e0009725. doi: 10.1371/journal.pntd.0009725 (PMC8489727; doi:10.1371/journal.pntd.0009725)
Supplement: S1 Table — (DOCX) [file pntd.0009725.s007.docx]

**S1 Table:** The comparison of the mean intensity, number of MS peaks and the signal to noise from *Bi. pfeifferi* head (H2) and foot (F2).

|  | ***Biomphalaria pfeifferi*** | |
| --- | --- | --- |
|  | Protocol F | Protocol H2 |
| **Mean intensity [a.u.]** | 173.0325 | 224.0342 |
| **The number of MS peaks** | 74 | 87 |
| **Signal to noise threshold** | 5.00 | 5.00 |
