# Supplementary material for: MALDI-TOF mass spectrometry for the identification of freshwater snails from Senegal, including intermediate hosts of schistosomes
Source: PLoS Negl Trop Dis. 2021 Sep 13;15(9):e0009725. doi: 10.1371/journal.pntd.0009725 (PMC8489727; doi:10.1371/journal.pntd.0009725)
Supplement: S2 Table — (DOCX) [file pntd.0009725.s008.docx]

**S2 Table:** *16S* partial sequences obtained from species that were morphologically identified as *Be. unicolor* and *C. bulimoides*.

| **Morphological identification** | **Number of specimens** | **BLAST results** | **Accession Number** | **Percentage ID** |
| --- | --- | --- | --- | --- |
| *Bellamya unicolor* | 2 | *Bellamya jeffreysi* | FJ405702.1 | 96.85% |
|  |  |  |  | 97.03% |
| *Cleopatra bulimoides* | 1 | *Cleopatra johnstoni* | KF412769 | 95.71% |
