# Supplementary material for: MALDI-TOF mass spectrometry for the identification of freshwater snails from Senegal, including intermediate hosts of schistosomes
Source: PLoS Negl Trop Dis. 2021 Sep 13;15(9):e0009725. doi: 10.1371/journal.pntd.0009725 (PMC8489727; doi:10.1371/journal.pntd.0009725)
Supplement: S3 Table — (DOCX) [file pntd.0009725.s009.docx]

**S3 Table:** Comparison of the mean intensity and the number of MS peaks of four specimens per species (*Bi. pfeifferi*, *Bu. truncatus* and *Bu. forskalii*) stored at -20°C and in ethanol.

|  | ***Biomphalaria pfeifferi*** | | ***Bulinus forskalii*** | | ***Bulinus truncatus*** | |
| --- | --- | --- | --- | --- | --- | --- |
|  | Frozen | Ethanol stored | Frozen | Ethanol stored | Frozen | Ethanol stored |
| **Mean intensity [a.u.]** | 220.6088 | 194.1681 | 219.4269 | 83.1450 | 204.3000 | 134.1450 |
| **The number of MS peaks** | 102 | 83 | 99 | 69 | 91 | 42 |
