# Supplementary material for: MALDI-TOF mass spectrometry for the identification of freshwater snails from Senegal, including intermediate hosts of schistosomes
Source: PLoS Negl Trop Dis. 2021 Sep 13;15(9):e0009725. doi: 10.1371/journal.pntd.0009725 (PMC8489727; doi:10.1371/journal.pntd.0009725)
Supplement: Attachment — Submitted filename: Hamlili_Author responses_June.docx [file pntd.0009725.s010.docx]

>Ms. Ref. No.:  PNTD-D-20-02023R1

>Title: MALDI-TOF mass spectrometry for the identification of freshwater snails from Senegal, including intermediate hosts of schistosomes.

>Running title: Identification of freshwater snails using MALDI-TOF MS

>PLOS NTD

**Reviewer #1 comments**:

**I/Methods:**
**1- A comparison between the number of peaks, intensity, signal to noise and reproducibility (CVs) using the snails' foot and the head should be provided.**

We would like to thank the reviewer for this important comment, and we understand the reviewer's concern regarding the protocol optimization. As requested, we compared the number of peaks, intensity, signal to noise for the head and the foot which are presented in the table below. We highlighted that the mean intensity and number of peaks generated from *Bi. pfeifferi* head (mean intensity: 224.0342, number of peaks: 87) are higher than those generated from the foot (mean intensities: 173.0325, number of peaks: 74). Besides, the signal to noise threshold for both body parts (the head and the foot) equals 5.00 as presented in the table below. For the CVs of both body parts, we provided the results in excel format. To bring more robustness to the analyses, we used the Composite Correlation Index (CCI) tool (MALDI-Biotyper v3.0. software, Bruker Daltonics), in order to assess spectra variations (MS spectra reproducibility) within each sample group according to the protocol tested. The mean CCI of protocol H2 was slightly higher (mean±SD: 0.88±0.08) than the protocol F (mean±SD: 0.84±0.13). In the results section, we have emphasized the reason of the selection of the foot for MALDI TOF MS analyses because the foot generally harbours the parasite and could potentially be used for the detection of *Schistosoma*. As reported, the primary sporocysts generally occur in the foot. This study opens a new avenue of research to a future project on the assessment of MALDI-TOF MS to differentiate between *Schistosoma*-infected from uninfected specimens. For more clarification, we added the following paragraph explaining the obtained results:

-Materials and Methods section**: Line 189-196”** **The Composite Correlation Index (CCI) tool (MALDI-Biotyper v3.0. software, Bruker Daltonics), was used to assess spectra variations within each sample group according to the protocol tested, as previously described [43]. CCI was computed using the standard settings of the mass range 3000–12000 Da, resolution 4, 8 intervals and autocorrelation off. Higher correlation values (expressed by mean ± standard deviation [SD]) reflect higher reproducibility of MS spectra (A CCI match value of 1 represents complete correlation, whereas a CCI match value of 0 represents an absence of correlation) [43]. The MS peaks and the intensity calculation of both the head and the foot were analysed using ClinProTools software”.**

-Results section**: Line 278-285 “To bring more robustness to the analyses, MS spectra reproducibility for each protocol was objectified using CCI matrix. The highest CCI (Fig 3C) was obtained for protocol H2 (mean±SD: 0.88±0.08) and protocol F (mean±SD: 0.84±0.13) than other protocols. The comparison of the number of MS peaks and intensity of specimens corresponding to the head (H2) and (F) showed that the mean intensity and number of MS peaks generated from *Bi. pfeifferi* head (mean intensity: 224.0342, number of MS peaks: 87) are higher than those generated from the foot (mean intensities: 173.0325, number of MS peaks: 74) (S1 table)”.**

**-**The legend section**: Line 1040-1041 “****Supplementary table 1: The comparison of the mean intensity, number of MS peaks and the signal to noise from *Bi. pfeifferi* head (H2) and foot (F2).”**

|  | ***Biomphalaria pfeifferi*** | |
| --- | --- | --- |
|  | Protocol F | Protocol H2 |
| **Mean intensity [a.u.]** | 173.0325 | 224.0342 |
| **The number of MS peaks** | 74 | 87 |
| **Signal to noise threshold** | 5.00 | 5.00 |

**2-   The authors wrote: “The difference between these protocols is not only in terms of the body parts used but also on the extraction solution volume (70% formic acid, 50% acetonitrile) and the homogenisation method (glass powder or tungsten beads)”. Are these two different solutions or the authors used a mixture of 50% acetonitrile and 35% formic acid and 15% water? This should be better clarified.**

The reviewer raised an important question regarding the volume of the extraction solution. The extraction solution consisted of 70% formic acid, 50% acetonitrile for all protocols, the difference was in the amount (i.e. volume) of the extraction solution used for grinding. We used either 30 µl or 40 µl of extraction solution as volume. We have clarified this in the materials and methods section as follows**: Line 150-157”** **We tested five different protocols using only two body parts (the head and/or the foot), homogenized in an extraction solution composed of a mix (50/50) of 70% (v/v) formic acid (Sigma-Aldrich, Lyon, France) and 50% (v/v) acetonitrile (Fluka, Buchs, Switzerland), according to the standardized automated setting previously described [37-39]. The difference between these protocols is not only in terms of the body parts used but also in the extraction solution volume (30 μl or 40 μl) and the** **homogenisation method: glass beads ≤ 106µm (Sigma Aldrich, Saint-Louis, Missouri, USA) or tungsten beads (Qiagen GmbH, Hilden, Germany) (Table 1)”**

**3-   Why the authors used this composition of the extraction solution and did not optimized the ratio acetonitrile/water to observe the difference in protein extraction? It is not reported the optimization of extraction solution based on the organic solvent type, concentration and type of acid.**

Thank you for this comment. Regarding the composition of the extraction solution, we used the extraction solution composed of 70% formic acid, 50% acetonitrile that was standardized and validated for arthropods as it allows a good extraction of proteins. Therefore, we did not change the composition of this extraction solution in our study. The suggestion of the reviewer is important, so the impact of the variation based on the ratio acetonitrile/water, the organic solvent type, concentration and type of acid will be evaluated on the extraction of proteins in future studies. In the materials and methods section, we reformulated the sentence as follows:

**Line 150-153: We tested five different protocols using only two body parts (the head and/or the foot), homogenized in an extraction solution composed of a mix (50/50) of 70% (v/v) formic acid (Sigma-Aldrich, Lyon, France) and 50% (v/v) acetonitrile (Fluka, Buchs, Switzerland), according to the standardized automated settings previously described [37-39].**

**4-   The 15, 30 and 40ul of extraction solution were optimized based on the weight of snails’ foots and heads? This point should be clarified.**

Thank you for this comment. The extraction solution volume was not based on the weight of the snail's foot and heads. However, the volume of the extraction solution is an important parameter to have spectra of good quality and as previously reported on arthropods (Sevestre *et al*., 2021; Benyahia H *et al.,* 2021) that the extraction solution volume differs from one arthropod to another and from one part of the same arthropod to another. For this reason, we have varied the volume of the extraction solution in order to select the volume that allows us to obtain quality and reproducible spectra. We take in account the reviewer’s helpful remark, and we are aware that it would have been interesting to weigh the body part used for the MALDI-TOF MS. However, in the coming projects on the detection of schistosomes within intermediate hosts by MALDI-TOF MS, it will be crucial to refine the protocol by weighing the foot tissue. For more clarification, we have added a sentence in the materials and methods as follows**: Line 161-162“****The different protocols tested had several variables including the volume of the extraction solution** **in order to select the optimal volume for protein extraction”.**

In the discussion section: **Line 438-447 “****The volume of the extraction solution is an important parameter to have spectra of good quality and as previously reported on arthropods [39,78] that the extraction solution volume differs from one arthropod to another and from one part of the same arthropod to another. For this reason, we have varied the volume of the extraction solution in order to select the volume that allows getting good quality and reproducible spectra. In our work, the optimisation of the extraction solution volume was not based on the weight of the snail’s foot and head tissue. However, for more precision, in future projects on the detection of schistosomes within the intermediate hosts by MALDI-TOF MS, it will be crucial to refine the protocol by taking into consideration the weight of the snail’s foot tissue.**

**-Citations:**

-Sevestre J, Diarra AZ, Laroche M, Almeras L, Parola P. Matrix-assisted laser desorption/ionization time-of-flight mass spectrometry: an emerging tool for studying the vectors of human infectious diseases. Future Microbiol. 2021 Mar;16:323-340. doi: 10.2217/fmb-2020-0145. Epub 2021 Mar 18. PMID: 33733821.

-Benyahia H, Ouarti B, Diarra AZ, Boucheikhchoukh M, Meguini MN, Behidji M, et al. Identification of Lice Stored in Alcohol Using MALDI-TOF MS. J Med Entomol. 2021;58: 1126–1133. doi:10.1093/jme/tjaa266

**5 -   The authors should better specify that the different protocols tested had several variables: 1) volume of extraction solution, 2) homogenization beads, 3) snail parts and 4) snail species.**

We thank the reviewer for this important remark. For more clarification, we have added the different parameters as suggested by the reviewer in the materials and methods section as follows: **line 161-164** “**The different protocols tested had several variables including the volume of the extraction solution in order to select the optimal volume for protein extraction, the homogenisation beads, the parts of the snail used and the snail species. All information about the protocols is summarized in table 1”.**

**6-   The authors state: “the choice of the body part that provides greater percentage of protein, and the simplicity of the protocol.”. The “greater percentage of proteins” should be changed in number of MS peaks.**

This comment from the reviewer made us realize that we have not explained this properly at all; we made all the necessary changes to avoid any ambiguity. In fact, prior to protocol development, we searched in the scientific literature which part of the snail’s body part was richer in proteins than other tissues, in an attempt to select the right body part for MALDI-TOF MS analysis. The foot was reported to be a good protein source in snails compared to other tissues (İhsan EKİN *et al*., 2016) and it happens to be where the primary sporocysts of *Schistosoma* generally occurs (Appleton C & Miranda N. 2015). MALDI-TOF MS has been used on mosquito cephalothoraxes to detect *Plasmodium* parasites so it made sense to select the snail foot for future attempt to detect *Schistosoma* parasites. Afterwards, we realized that indeed this body part provides enough proteins for MALDI-TOF MS analysis and good quality spectra. It was therefore selected for further analyses. We clarified as follows in the materials and methods section:

**-Line 145-146: The snail’s foot was selected for MALDI-TOF MS protocol optimisation because it was stated to be a good source of proteins than other tissues [36].**

**-Line 159-161:** **the protocols were assessed initially on the basis of intraspecies reproducibility, spectra intensity** **≥ 3.000 arbitrary units [a.u.], the number of MS peaks and the simplicity of the protocol.**

-In the discussion section:

**Line 427-433:** **The findings showed that MALDI-TOF MS spectra obtained from the snail foot (F), head (H) and foot-head (FH) protein extracts were adequate to accurately identify** **freshwater snails involved in schistosomiasis transmission.** **The protocol validation was confirmed using only the foot. Precisely, we selected the foot for sample preparation for two reasons, firstly the primary sporocysts of *Schistosoma* generally occur in the foot and secondly, it was reported that this snail’s body part was a good protein source compared to other snail tissues.**

**-Citations:**

**-** Appleton C, Miranda N. Locating bilharzia transmission sites in South Africa: guidelines for public health personnel. South African J Infect Dis. 2015;30: 95–102. doi:10.1080/23120053.2015.1074438

-Ekin İ, Şeşen R, Alkan H, Akbal E, Başhan M. Protein content of five tissues from some edible and non-edible snails and bivalves distributed within turkish territories. Soc Sci Nat. 2016;6: 435–438. doi:10.3329/jsr

**7-   A saturated solution of alpha-cyano-4-hydroxycinnamic acid dissolved in 50% acetonitrile (v/v), 10% trifluoroacetic acid (v/v) was used. Saturated means 10 mg/mL? For method optimization, did the authors test different matrices such as sinapinic acid dissolved in different concentration of acids? This point should be addressed.**

**-Response to “A saturated solution of alpha-cyano-4-hydroxycinnamic acid dissolved in 50% acetonitrile (v/v), 10% trifluoroacetic acid (v/v) was used. Saturated means 10 mg/mL? “**

Thank you for pointing out this comment. In our study, we did not particularly measure how saturated the matrix is, but it was very saturated, higher than 10mg/ML. We put a large amount of the matrix (alpha-cyano-4-hydroxycinnamic acid) compared to the mix of acetonitrile and trifluoroacetic acid, so that the solution is excessively cloudy after sonication. After that, we centrifuge the solution and we recover the supernatant.

Concerning the TFA concentration. We do not use different concentration of acids, it is in fact, it is an error in the paper. We used 2.5% TFA as cited in reference 40, 41 and 42. This has now been amended to avoid ambiguity and we clarified as follows. **Line 177-179:** **matrix suspension composed of saturated alpha-cyano-4-hydroxycinnamic acid (Sigma, Lyon. France), 50% acetonitrile (v/v), 2.5% trifluoroacetic acid (v/v) (Aldrich, Dorset, UK) and HPLC grade water [40-42]**

**-Response to” For method optimization, did the authors test different matrices such as sinapinic acid dissolved in different concentration of acids? This point should be addressed”**

The reviewer is right. We did not test different matrices such as sinapinic acid. We only used alpha-cyano-4-hydroxycinnamic acid, which has been successfully used in several protocols for arthropods identification as well as it has shown its performance in clinical microbiology. In particular, the identification of many microorganisms such as, mycobacteria, bacteria, yeasts, and fungi. Besides, the spectra obtained using this matrix used in our study were of sufficient quality. Therefore, we validated the use of alpha-cyano-4-hydroxycinnamic acid for matrix preparation in order to identify snails. In case, we had failed to get good quality spectra, we would have tested different matrices based on sinapinic acid and we would have tested different concentrations. For more clarification, we added in the discussion section the following paragraph: **Line 413-424”** **As for the matrix preparation, we used alpha-cyano-4-hydroxycinnamic acid which has been successfully used for several arthropods identification [27,40,43,66]. As well, it has shown its performance in clinical microbiology. In particular, the identification of many microorganisms such as mycobacteria, bacteria, yeasts, and fungi [72–75]. Besides, the use of this matrix was sufficient to yield spectra of high quality of different snail species. Therefore, we validated the use of alpha-cyano-4-hydroxycinnamic acid for matrix preparation. Otherwise, we would have tested different acids as sinapinic acid in case we have failed to get good signals with the best resolution. The use of such matrix preparation and glass beads have previously shown their performance and robustness in different conditions [39,40,69–72]. Consequently, in the present work, the combination of glass beads and matrix based on alpha-cyano-4-hydroxycinnamic acid revealed promising results for freshwater snail identification.”**

**8- The MALDI-TOF data should be deposited in a public repository such as PRIDE mass spectrometry data repository since they constitute a useful resource for the community.**

The reviewer is right. Our database is currently accessible on demand through collaboration with other researchers, and we are working to deposit the spectra in a suitable public repository tool. We mentioned this in the data availability section as follows: **Line 623-625 “Our MALDI TOF-MS snail data are currently accessible on demand through collaboration with other researchers meanwhile we are working to deposit the spectra in a suitable public repository tool [99]**.”

**II/ Results**

**1-   In Figure 3, the authors should clearly define the MS parameters to define the best extraction protocol.**

The reviewer observations are exact. The MS parameters were added in the legend of Figure 3 as suggested. **Line 998-999**: **The MS parameters:** **intensity ≥ 3.000 [a.u.], absence of background noise and the reproducibility of MS spectra.**

**2-   A direct comparison between MALDI-TOF MS spectra obtained from frozen and ethanol-stored samples should be performed to evaluate the effect of sample storage conditions on the spectra quality.**

We thank the reviewer for this comment. To show a comparison between MS spectra from frozen and ethanol-stored specimens. We used 4 specimens from each species (*Bi. pfeifferi*, *Bu. truncatus*, *Bu. forskalii*) for both storage conditions in order to create a dendrogram using MALDI-Biotyper software v.3.0 and run a PCA (principal component analysis) with ClinProTools software. In addition, we calculated the mean of peaks intensity and the number of peaks of the MS spectra to visualize the impact of preservation method on the spectra quality.

The principal component analysis (S3 Fig) showed a clear distinction between frozen, and ethanol-stored specimens for each species: *Bi. pfeifferi*, *Bu. truncatus*, *Bu. forskalii*. The comparison of MS spectra of both storage conditions using a dendrogram also showed that the MS spectra of frozen specimens of each species (*Bi. pfeifferi*, *Bu. truncatus* and *Bu. forskalii*) clustered separately from ethanol stored MS spectra of the same species (S4 Fig). Accordingly, a specific database is necessary for each storage condition in order to get a correct identification for frozen and ethanol stored specimens of each species. In the obtained dendrogram (S4 Fig), we noticed that the MS spectra of frozen specimens were so different from the ones in alcohol of the same species that they were positioned on another branch completely different from the frozen ones and in some cases, they were so different that they were closer to another species. Particularly, in this case, frozen *Bu. truncatus* MS spectra were closer to MS spectra of *Bu. forskalii*. Consequently, this reflects a deep difference between these two storage conditions.

In order to bring more robustness to the results obtained by dendrogram and PCA, we provided the mean intensity and the number of peaks of each species (n=4) stored in both storage conditions which is presented in the table below. The peak intensities of frozen specimens from each species were higher than ethanol stored specimens which presumes the effect of ethanol on the intensities of spectra. As well, we noticed that the number of peaks was higher in frozen specimens for the three species mainly in *Bu. truncatus*. Here, we see the effect of ethanol on both the intensities and the number peaks. To sum up, the frozen storage method appears to be the best for preservation for MALDI-TOF MS analyses. Besides, the impact of alcohol on the quality of the spectra has been already reported for ticks by Diarra *et al.* (2017), Kumsa *et al.* (2016), for fleas by Zurita *et al.* (2019) and Nebbak *et al.* (2017) and also for cercariae by Huguenin *et al.* (2019). Therefore, the creation of a specific database of ethanol stored as well as frozen snails is important for a reliable identification. For more clarification, we have made changes in the document to highlight the impact of alcohol on the quality of the spectra. In the materials and methods section, we have added this sentence: **line 206-211 “****To evaluate the impact of the storage method on the quality of the MS spectra, a comparison of MS spectra of 4 specimens from each species (*Bi. pfeifferi*, *Bu. truncatus*, *Bu. forskalii*) preserved either in ethanol or at -20°C was performed using the MS dendrogram (MALDI-Biotyper v.3.0) and the principal component analysis (ClinProTools software). The mean of peaks intensity and the number of peaks were also calculated for these spectra”.**

-In the Result section we added the following sentences**: in line 328-****343 “****The principal component analysis (S3 Fig) unveiled a clear distinction between the MS spectra of specimens of the same species preserved either at -20°C or in ethanol. The MS spectra comparison of both storage conditions using a dendrogram also showed that the MS spectra of frozen specimens of each species (*Bi. pfeifferi*, *Bu. truncatus* and *Bu. forskalii*) clustered separately from ethanol-stored MS spectra of the same species (S4 Fig). Accordingly, a specific database is necessary for each storage condition in order to get a correct identification for frozen and ethanol-stored specimens of each species. The mean spectra intensities and the number of MS peaks of *Bi. pfeifferi*, *Bu. truncatus* and *Bu. forskalii* were higher for frozen specimens than ethanol-stored specimens of the same species. All the obtained values of MS spectra intensities and the number of MS peaks are presented in Table S3. In the obtained dendrogram (S4 Fig), we noticed that the MS spectra of frozen specimens were so different from the ones in alcohol of the same species that they were positioned on another branch completely different from the frozen ones and in some cases, they were so much different that they were closer to another species. Particularly, in this case, frozen *Bu. truncatus* MS spectra were closer to the MS spectra of *Bu. forskalii*. Consequently, this reflects a deep difference between these two storage conditions.**

-In the discussion section we have added the following sentence**: Line 541-546 “****On the other hand, we noticed differences at the MS profiles level of both frozen and ethanol-stored specimens of the same species. Specifically, the mean spectra intensities and the number of MS peaks of frozen specimens of *Bi. pfeifferi*, *Bu. truncatus* and *Bu. forskalii* were higher than those of specimens of the same species stored in ethanol (S3 Table). This difference may be due to the effect of** **ethanol on the quality of the spectra as previously reported in ethanol stored ticks, fleas and cercariae.**

-In the legend section:

**Line 1033-1039**

**Supplementary data figure 3: MALDI-TOF MS spectra distinction from frozen and ethanol-stored *Bi. pfeifferi*, *Bu. forskalii*, *Bu. truncatus* by principal component analysis using ClinProTools 2.2. (A) *Bi. pfeifferi*, (B) *Bu. forskalii*, (C) *Bu. truncatus*.**

**Supplementary data figure 4: Comparison of MALDI-TOF MS spectra of frozen and ethanol-stored from four specimens of three species: *Bi. pfeifferi*, *Bu. truncatus* and *Bu. forskalii* using a dendrogram (Biotyper software v.3.0). Frozen specimens are indicated in Bold and ethanol-stored specimens are not in Bold.**

**Line 1044-1046 “Supplementary table 3: Comparison of the mean intensity and the number of MS peaks of four specimens per species (*Bi. pfeifferi*, *Bu. truncatus* and *Bu. forskalii*) stored at -20°C and in ethanol.**

|  | ***Biomphalaria pfeifferi*** | | ***Bulinus forskalii*** | | ***Bulinus truncatus*** | |
| --- | --- | --- | --- | --- | --- | --- |
|  | Frozen | Ethanol  stored | Frozen | Ethanol stored | Frozen | Ethanol stored |
| **Mean intensity [a.u.]** | 220.6088 | 194.1681 | 219.4269 | 83.1450 | 204.3000 | 134.1450 |
| **The number of MS peaks** | 102 | 83 | 99 | 69 | 91 | 42 |

**3-   The authors should compare the MALDI-TOF MS spectra from** **Schistosoma-infected snails to the uninfected ones.**

We appreciate the reviewer’s insightful suggestion and agree that it would be useful to compare MS spectra of infected specimens from uninfected ones. However, we could not compare MALDI-TOF MS spectra from *Schistosoma*-infected snails to the uninfected because the number of infected specimens was not enough to create a database for *Schistosoma*-infected snails and perform a blind test. It was therefore not possible to validate the results. Moreover, for the same reason, we could not perform a robust statistical analysis using the principal component analysis (PCA). For *Bi. pfeifferi*, we obtained only one specimen infected, for *Bu. truncatus*, three specimens were infected (of the three specimens, 2/3 spectra of good quality were obtained for *Bu. truncatus*) and only one specimen of *Bu. senegalensis* was infected. Nevertheless, we mentioned in the perspectives that further studies will be conducted to assess the utility of MALDI-TOF MS to distinguish infected from uninfected specimens. As well, we plan to do it in a future project. The paragraphs are mentioned in the discussion as follow:

-In the result section: **Line 388-391 “The determination of the infectious status based on the comparison of MS spectra of both infected and uninfected specimens belonging to *Bi. pfeifferi*, *Bu. truncatus* and *Bu. senegalensis* was not performed because of the low number of infected specimens.**

-In the discussion section: **Line** **548-554** “**Unfortunately, we could not compare MALDI-TOF MS spectra from *Schistosoma*-infected snails to the uninfected ones because the number of infected specimens was not enough to create a specific database for *Schistosoma*-infected snails and also perform a blind test in order to validate the results. Moreover, we could not perform a robust analysis using the principal component analysis (PCA) to distinguish infected and non-infected specimens because of the low number of MS good quality spectra from infected specimens; *Bi. pfeifferi* (n=1), *Bu. truncatus* (n=2), and *Bu. senegalensis* (n=1).”**

**Line 572-576: Provided that the MALDI TOF MS protocol has been standardized, it is necessary to assess the ability of MALDI TOF MS to detect the infection within the intermediate hosts either by artificially infecting the freshwater snails (*Biomphalaria* or *Bulinus*) or collecting snails in *Schistosoma*-endemic areas in an attempt to help mapping the risk of disease transmission or to set-up long-term control strategies.**

    --------------------

**Reviewer #2 comments:**

**1-Abstract line 28-29: “…has revolutionized clinical microbiology and mycology…”, mycology is a part of clinical microbiology.**

Thank you for this remark. The correction has been made as suggested.

**Line 28-29: MALDI-TOF MS has revolutionised clinical microbiology and has emerged in the medical entomology field.

2-Author summary line 48: “…blood flukes of the genus Schistosoma which are released by freshwater snails…” only the cercarial stage is released.**

This observation is correct. We have made the change. The new sentence reads as follows:

**Line 47-48: Schistosomiasis is a parasitic disease, caused by blood flukes of the genus *Schistosoma*. The infective cercariae are released by freshwater snails belonging to three genera**… **3-Ref 8: is a French classic, but it is now a bit outdated. Recent data on Schistosomiasis epidemiology can be found in the SCORE special issue of Am. J. Trop. Med. Hyg. (N° 103 Suppl1,2020).**

The outdated reference has been changed with the suggested new reference: **Line 68** **[8] Colley DG, Fleming FM, Matendechero SH, Knopp S, Rollinson D, Utzinger J, et al. Contributions of the schistosomiasis consortium for operational research and evaluation (SCORE) to schistosomiasis control and elimination: Key findings and messages for future goals, thresholds, and operational research. Am J Trop Med Hyg. 2020;103: 125–134. doi:10.4269/ajtmh.19-0787**

**4-line 83: B. umbilicatus**

The suggested correction has been made. **Line 83: *Bu. umbilicatus***

**5-line 90: I suggest “depend mainly” or “relies mainly on” instead of “often depends”.**

The text has been revised as suggested: **line 90: snail classification relies mainly on morphological criteria.**

**6-line 98-99: “MALDI-TOF (Matrix Assisted Laser Desorption / Ionization Time-of-Flight) mass spectrometry” could be changed to** **MALDI-TOF MS (Matrix Assisted Laser Desorption / Ionization Time-of-Flight Mass Spectrometry) as MALDI-TOF MS is the abbreviation used.**

The correction has been made as suggested. **Line 98-99: MALDI-TOF MS (Matrix Assisted Laser Desorption / Ionization Time-of-Flight Mass Spectrometry)**

**7-line 108: Stephan et al. (2) were the first to report MALDI-TOF use for specific identification in Mollusca.**

We thank the reviewer for this important remark. **Line 103-105: To the best of our knowledge, this innovative tool has been firstly used for the identification of scallops [30], but it has not been yet applied to identify snails of medical importance.**

**[30] Stephan R, Johler S, Oesterle N, Näumann G, Vogel G, Pflüger V. Rapid and reliable species identification of scallops by MALDI-TOF mass spectrometry. Food Control. 2014;46: 6–9. doi:10.1016/j.foodcont.2014.04.047”**

**8-line 118: “The weather is warm all year round.” This sentence is not informative as average, and min/max are given in the next sentence.**

As suggested, the following sentence: the weather is warm all year round, has been removed from **line 117**.

**9-line 151: “extraction solution proportion” instead of “volume”?**

The reviewer is right. It is in fact an error in the paper, we kept the sentence “extraction solution volume” because we wanted to mention the volume **30 μl or 40 μl** and not the extraction solution proportion (70% formic acid, 50% acetonitrile). The error has been fixed. **Line 153-155: The difference between these protocols is not only in terms of the body parts used but also in the extraction solution volume (30 μl or 40 μl) and the homogenisation method.**

**10-Line 351: It could be interesting to cite the paper by Stephan et al. (2) as it is the first to report MALDI-TOF use for specific identification in Mollusca.**

We wish to thank the reviewer for bringing this paper to our attention. The citation has now been added as suggested. **Line 401-403: this proteomic approach has also been applied to malacology as Stephan *et al.* was the first to report the MALDI-TOF MS use for identification of marine bivalve molluscs specifically the scallops [30]**.

**11-Line 339: “We performed a qPCR for the detection of S. mansoni and S. haematobium” Interestingly, the probes from Cnops et al. and the Sh-Fw and Sh-Rv primers, could hybridize with other species of the S. haematobium complex, thus S. intercalatum or S. currassoni are detected. I propose to change to “****We performed a qPCR for the detection of S. mansoni and S. haematobium species complex”.**

Thank you for this remark, this has been corrected as suggested. **Line 384: We performed a qPCR for the detection of *S. mansoni* and *S. haematobium* species complex**

**12-line 259-260: “an unsupervised test” instead of “the unsupervised test”**

The suggested correction has been made. **Line 274:** **an unsupervised statistical test**

**13-line 270: “as confirmed by Flex Analysis”, I suppose by visual inspection?**

The reviewer is right. The data was confirmed by visual inspection. **Line 270: By visual inspection**

**14-line 360: “adequate” instead of enough?**

The suggested correction has been made. **Line 428:** **adequate
15-Table 2: M. tuberculata instead of “Melanoides tuberculate”, or provide full-name for all species.**

We thank the reviewer for this remark. The error has been fixed. *M. tuberculata*. We kept the abbreviation**. Line 1061 “(Table 2): *M. tuberculata* “**

**16-Table 3: “Good quality Spectra” instead of “Good Spectra”**

The correction has been made as suggested. **Line 1063: good quality spectra**

**17-Table 3: For the fifth column, did the percentage correspond to percentage of similarity against best match in Genbank? Please precise in the column title.**

Yes, the percentage corresponds to the Identity percentage. I added % of identity. % of identity is an abbreviation of identity percentage. **Line 1061-1063 (table 2 and 3): % of identity**

**18-Table 3: Please provide a foot-note explanation of the character “*” (as in Table 2 legend)**
The correction has been made as suggested. **Line 1062 and 1064: *Discrepancy between morphological and molecular identification.**

**19-Figure 3 panel B: To which Principal Component correspond x and y axis of the 2D plot?**

Thank you for your interesting comment. The PC1 is the x-axis and PC2 is the y-axis. In the figure 3, we replaced the principal component Analysis in 2D with 3D because the data in 3 dimensions are more visible. To bring more robustness to the analysis, we calculated the explained variance of PC1, PC2 and PC3 and we have added a paragraph on the explained variance in the results section: **Line 276-278 “Using ClinProtools, the contributions of PC1, PC2, and PC3 to the generation of profile in a percentage the plot of the variance explained were approximately 38.18%, 16.81%, and 11.36%, respectively as presented in the following S1 Figure”.**

The legend**: Line 1023-1027 “Supplementary data figure 1: The dimensional image from PCA indicating the comparison of MALDI-TOF MS spectra from five different protocols (H1, H2, H3, FH, F) using frozen *Bi. pfeifferi*. The figure shows the contribution of ten principal components to the profiling classification in plot of percentage explained variance of PC. Red dots: protocol H1, green dots: protocol H2, blue dots: protocol H3, yellow dots: FH and purple dots: F.”**

**20-Figure 5 panel A: To which Principal Component correspond x and y axis of the 2D plot? The variance explained by each component could also be an interesting data.**

The PC1 is the x-axis and PC2 is the y-axis. As suggested by the reviewer, we calculated the explained variance of PC1, PC2 and PC3. We have added a paragraph on the explained variance in the results section **Line 314-316: “****Using ClinProtools, the contributions of PC1, PC2, and PC3 to the generation of profile in a percentage plot of the variance explained were approximately 35.65%, 26%, and 17.39%, respectively as presented in the following S2 Figure”.**

**Line 1015: The x-axis represents PC1 and the y-axis represents PC2.**

The legend**: Line 1028-1032 “Supplementary data figure 2: The dimensional image from PCA indicating the comparison of MALDI-TOF MS spectra from the foot of ethanol-stored *Bi. pfeifferi* specimens belonging to three different protocols. The figure shows the contribution of eight principal components to the profiling classification in plot of percentage explained variance of PC. Red dots: protocol F1, green dots: protocol F2, blue dots: protocol F3.”**

    --------------------

-**The global design of the study is clear but the number of specimens used in the different groups is hard to follow. Please clarify the relevant parts in materiel and methods, results and table sections. To my opinion the better option is to provide a flowchart of the different experiments. For instance, in table 3 (identification results of ethanol-stored snails) the total of column “Specimen collection” is 210 but the addition of B. pfeifferi, B. truncatus and B. senegalensis specimen is 160. I understand with this table that 80 specimens were tested and 40 used for blind-tested. The use of the 40 remaining specimens is unclear to me. In parallel, the text refers to 244 ethanol-stored snails, minus 13 used for testing protocols.**

**A/Please clarify the relevant parts in materiel and methods, results and table sections. To my opinion the better option is to provide a flowchart of the different experiments.**

We are grateful for this important comment as it points to make the study clearer for readers. We did a flowchart as suggested to provide better clarification. The flowchart is represented in the Figure 2. The study design is divided into three sections: A/ protocol optimisation, B/database creation and C/ validation with Blind test.

-The explanation of the flowchart: during the study, two field collection missions were carried out; the first field collection was done in 2018 and the second field collection was carried out in 2019. We used the first field collection specimens for protocol optimisation and the second field collection specimens for protocol validation and database creation. Laboratory reared specimens (*Bulinus and Biomphalaria*) were also used for database creation and validation. As suggested, we summarized all the information regarding the study design, experiments, and the number of specimens in the flowchart. For more precision, we added the explanation of the flowchart in the legend. As well, we added in the material and methods section the following sentence: **Line** **213-214 “The design of the study is summarized in the flowchart represented in figure 2.”**

In the legend: **Line 986-990 “Figure 2:** **Flowchart showing the study design and all samples collected from the first and second field collection [2018-2019] and those used for each step in the study. The study design is divided into three sections: (A) protocol optimisation, (B) database creation and (C) the validation with a blind test. The number and the species of field and laboratory-reared freshwater snails are mentioned.”**

**B/For instance, in table 3 (identification results of ethanol-stored snails) the total of column “Specimen collection” is 210 but the addition of B. pfeifferi, B. truncatus and B. senegalensis specimen is 160.**

We thank the reviewer for pointing out this comment. It is in fact an error in our paper. We have made the correction in the table 3 (column specimen collection): **Line 1063 (table 3) “160 specimens”.** Additionally, we mentioned in the flowchart, the number of specimens used for the MALDI-TOF MS identification to make it more clear for readers.

**C/I understand with this table that 80 specimens were tested and 40 used for blind-tested. The use of the 40 remaining specimens is unclear to me.**

-The reviewer raised a question regarding the 40 specimens used for Blind test. For MALDI-TOF analysis, we tested in total 80 specimens from which we obtained 57 good quality spectra. Of the 57 spectra, we selected 17 spectra for database creation. The remaining spectra (40 spectra) were then used for Blind test. For more clarification to the reviewer, we created the following flowchart, and we added the explanation in the result section as follows:

**Line 319-320:** **Eighty specimens out of 160 were used to validate protocol F3 (Fig 2). Of 80 spectra analysed by Flex analysis, 57 spectra were of good quality.**

**Line 323-327: a specific database was created, comprising seventeen MS spectra: *Bi. pfeifferi* (n=9), *Bu. truncatus* (n=4), *Bu. forskalii* (n=2), *Bu. globosus* (n=1), and *Bu. senegalensis* (n=1), and the remaining 40 spectra were used for the blind test (Table 3). Of the 40 specimens, 22/22 *Bi. pfeifferi*, 3/10 *Bu. truncatus*, 4/6 *Bu. forskalii* and 2/2 *Bu. globosus* were correctly identified at the species level (77.50 %).**

80 specimens tested for MALDI TOF analysis

57 Spectra of good quality

17 spectra used for database creation

40 spectra were used for Blind test

23 Spectra of bad quality

**D/ For instance, in table 3 (identification results of ethanol-stored snails) the total of column “Specimen collection” is 210 but the addition of B. pfeifferi, B. truncatus and B. senegalensis specimen is 160. I understand with this table that 80 specimens were tested and 40 used for blind-tested. The use of the 40 remaining specimens is unclear to me. In parallel, the text refers to 244 ethanol-stored snails, minus 13 used for testing protocols.**

The reviewer is right. The 244 specimens mentioned in the text are from the two field collection missions. Of 244 specimens stored in ethanol, 84 specimens were from the first filed collection mission and 160 specimens were collected during the second field collection mission. However, 13/84 specimens were used for protocol optimisation and 80/160 were used for MALDI-TOF MS identification (protocol validation). For more clarification, we provided to the reviewer an exhaustive flowchart which summarizes the number of snails stored in ethanol used for protocol optimisation and for MALDI-TOF MS identification. As well, in the material and methods section, we added the following sentence**: Line 167-168 “****A second protocol was developed for ethanol-stored samples specifically. Of the 84 specimens preserved in ethanol, 13 were used for protocol optimisation”**

**Line 319: Eighty specimens out of 160 were used to validate protocol F3 (Fig 2).**

Second collection mission (n=160)

First collection mission (n=84)

Specimens stored in ethanol (n=244)

Specimens used for protocol optimisation

(n=13)

(n=13)

23 Spectra of bad quality

57 Spectra of good quality

17 spectra used for database creation

40 spectra were used for Blind test

80 specimens tested for MALDI TOF analysis

**-line 151: in order to reproduce the protocol, information regarding the amount of snail tissue is important. Was the volume of tissue the same as the mix volume?**

The reviewer raised a good point here. However, we did not measure the volume of the tissue. We used 30ul of extraction solution. Given the size (small) of the tissue of the freshwater snails tested, the volume of the mix volume was greater than the part of the body used (foot). We take in account the reviewer’s helpful remark, and we are aware that it would have been interesting to weigh the body part used for the MALDI-TOF MS. However, in the coming project on the detection of schistosomes within intermediate hosts by MALDI-TOF MS, it will be crucial to refine the protocol by weighing the foot tissue. For more clarification, we have added a sentence in the materials and methods as follows**: Line 161-162“The different protocols tested had several variables including the volume of the extraction solution in order to select the optimal volume for protein extraction”.**

In the discussion section: **Line 438-447 “The volume of the extraction solution is an important parameter to have spectra of good quality and as previously reported on arthropods [39,78] that the extraction solution volume differs from one arthropod to another and from one part of the same arthropod to another. For this reason, we have varied the volume of the extraction solution in order to select the volume that allows getting good quality and reproducible spectra. In our work, the optimisation of the extraction solution volume was not based on the weight of the snail’s foot and head tissue. However, for more precision, in future projects on the detection of schistosomes within the intermediate hosts by MALDI-TOF MS, it will be crucial to refine the protocol by taking into consideration the weight of the snail’s foot tissue.**

**Citations:**

-Sevestre J, Diarra AZ, Laroche M, Almeras L, Parola P. Matrix-assisted laser desorption/ionization time-of-flight mass spectrometry: an emerging tool for studying the vectors of human infectious diseases. Future Microbiol. 2021 Mar;16:323-340. doi: 10.2217/fmb-2020-0145. Epub 2021 Mar 18. PMID: 33733821.

-Benyahia H, Ouarti B, Diarra AZ, Boucheikhchoukh M, Meguini MN, Behidji M, et al. Identification of Lice Stored in Alcohol Using MALDI-TOF MS. J Med Entomol. 2021;58: 1126–1133. doi:10.1093/jme/tjaa266

**- line 339 The primer combinations used has not been tested with all other larval trematodes that could potentially infest the study snails.  Bulinus and Biomphalaria can be infested with** **Echinostoma or Paramphistomidae (3,4). According to Hamburger et al. (5), Sh-Fw/Sh-Rv don’t hybridize with Echinostoma. Were the ethanol preserved snails visually inspected during dissection to assess the absence or presence of trematodes? The infection with non-detected larval trematodes could introduce an important bias in the acquiredspectra.**  
We appreciate the reviewer’s insightful remark, and we understand the reviewer's concerns. In fact, in our study the ethanol stored snails have only been examined for *Schistosoma* cercarial shedding. No trematodes visual inspection has been done while the dissection. Nevertheless, we recognize this remark should be mentioned in the paper (Discussion), so we added the following sentence: **Line 558-571: “****Echinostomes and schistosomes are two genera of trematodes that utilize freshwater snails [93] as intermediate hosts. As stated by a recent study, echinostomes can infect some intermediate hosts of schistosomes, namely *Bulinus* and *Biomphalaria*, as their first intermediate host [94]. However, echinostomes were shown to be active competitors of schistosomes, limiting the ability of the latter to settle in the snail [95,96]. It was specifically reported that *E. caproni* limits the infection of *Bi. glabrata* by *S. mansoni* [97]. In our study, snail specimens belonging to *Biomphalaria* and *Bulinus* were screened for the presence of *Schistosoma* infection** **but these other parasites such as *Echinostoma* or Paramphistomidae [94,98] could infect the same species and may potentially impact the MS spectra. In future studies aimed at developing MALDI-TOF MS for the detection of schistosomes in snails, it would be** **interesting to better characterise the specimens and ensure the thorough investigation of parasites commonly associated with the tested snails. This current study is however focused on the application of MALDI-TOF MS technique in order to identify snail populations including the species that play an indispensable role in the schistosomiasis transmission.**

**-Please precise if the snail MALDI-TOF database is available on request.**

Our database is currently accessible on demand through collaboration with other researchers, and we are working to deposit the spectra in a suitable public repository tool. We mentioned this in the data availability section as follows: **Line 623-625 “Our MALDI-TOF MS snail data are currently accessible on demand through collaboration with other researchers meanwhile we are working to deposit the spectra in a suitable public repository tool [99]**.”

**Minor remarks:**

**-line 173: the composition of the commercial Bruker standard solvent (OS solution) for resuspension is:  Acetonitrile 50%, trifluoroacetic acid (TFA) 5%, Water 45% (Sigma ref 900666). In ref 34 the composition of OS solution is the same as in the present paper but it cite another reference from Marseille (1) with 2.5% of TFA. What is the purpose of increasing the proportion of TFA?** 
We thank the reviewer for pointing out this comment. In fact, it is an error in the paper. In our study, we used 2.5% TFA as cited in ref 40, 41 and 42. We apologize for our error; it has been fixed. **Line 177-179:** **50% acetonitrile (v/v), 2.5% trifluoroacetic acid (v/v) (Aldrich, Dorset, UK) and HPLC grade water [40-42]**

**- line 305: Did you succeed to obtain sequence for all specimens?**

Thank you for this comment. For frozen specimens, we selected 27 specimens for database creation, and we successfully obtained 27 sequences of high quality of different species using *COI* gene. For ethanol-stored specimens, we obtained *COI* sequences for 24 specimens belonging to species of the genus *Biomphalaria* and *Bulinus*: 17 were used for database creation and the remaining specimens (n=7) were used for morphological identification. Additionally, we used the *16S* gene to confirm the molecular identification of 2 specimens of *Be. unicolor* and one specimen of *C. bulimoides*. In supplementary data figure 5, we will attach all the accession numbers and the FASTA of *COI* and *16S* gene.

We clarified this in the manuscript as follows:

In the result section: **In line 345-348: “****Twenty-seven specimens were selected for database creation and morphological identification. We successfully obtained 27 sequences of high quality of different species using *COI* gene****: 9 *Bu. truncatus*, 6 *Bi. pfeifferi*, 4 *Bu. senegalensis*, 3 *L. natalensis*, 2 *Be. unicolor*, 2 *M. tuberculata* and 1 *C. bulimoides*.**”

**In line 360**-**362: “****For ethanol-stored specimens, we obtained *COI* sequences for 24 specimens belonging to species of the genus *Biomphalaria* and *Bulinus*: 17 were used for database creation and the remaining specimens (n=7) were used for morphological identification.”**

**Line 375-380: In order to confirm the molecular identification of both specimens that had been morphologically identified as *Be. unicolor* and *C. bulimoides*, a fragment of the *16S* gene was sequenced. The BLAST results showed that sequences of *Be. unicolor* (n=2) matched with sequences of *Bellamya jeffreysi* (ID range score 96.85–97.03%: FJ405702.1) and the sequence of the *C. bulimoides* (n=1) was designated as** ***Cleopatra johnstoni* (ID score 95.71%: KF412769).**

**- Line 368 and line 812 table 1: Disruption using tungsten beads was not tested with Foot and Head-Foot in frozen specimens. What is the reason?**

The reviewer raised a good question. For the protocol optimization, the tungsten beads (H1) and the glass beads (H2) were considered as variable parameters while other parameters such as body part (the head) and the extraction solution proportion, were kept constant in the attempt to validate the most efficient disrupting agent for grinding. The results showed that the spectra generated by glass beads were much better in term of quality than spectra generated by tungsten beads. Therefore, we validated this parameter (glass beads) to be applied on the other body parts (foot and head-foot). In fact, for protocol optimisation, we tested these two grinding methods (tungsten beads and the glass beads) because both methods reportedly have proven to be useful and effective in previous studies. However, glass beads worked very well on different snail species identification since the MS spectra obtained were of good quality. Additionally, the glass beads are the most commonly used for various arthropod identification. As well, they were shown to be robust in sample preparation for the identification of mycobacteria as they are used to disrupt the mycobacterial cell wall. For fungal identification, the glass beads were the most efficient for fungal protein extraction compared steel beads since the latter lead to high degradation of fungal proteins.

We clarified in the discussion this point: **Line 406-413:** **“****we tested two kinds of disrupters: both tungsten beads and glass beads have proven to be useful and effective grinding methods in previous MALDI-TOF MS studies [40,66–68]. However, glass beads were reportedly the most commonly used disrupter, as they have been used for various arthropods identification [39,40,66], for mycobacteria identification, to disrupt the mycobacterial cell wall [69,70], and for fungal identification, as they were most efficient than steel beads for fungal proteins extraction [71]. The glass beads also appeared to be the most suitable for snail sample homogenisation, as they provided reproducible and intense MS spectra for snail species. “**

**References:**

**The references were added to the manuscript as suggested:**

    **[26].  Fournier P-E, Couderc C, Buffet S, Flaudrops C, Raoult D. Rapid and cost-effective identification of Bartonella species using mass spectrometry. J Med Microbiol. 1 sept 2009;58(9):1154‑9. 

    [30].  Stephan R, Johler S, Oesterle N, Näumann G, Vogel G, Pflüger V. Rapid and reliable species identification of scallops by MALDI-TOF mass spectrometry. Food Control. 1 déc 2014;46:6‑9. 

    [33].** **Chappell LH. Freshwater snails of Africa and their medical importance (second edition). Trans R Soc Trop Med Hyg. nov 1994;88(6):717.**

**[51].  Hamburger J, Abbasi I, Ramzy RM, Jourdane J, Ruppel A. Polymerase chain reaction assay based on a highly repeated sequence of Schistosoma haematobium: a potential tool for monitoring schistosome-infested water. Am J Trop Med Hyg. 2001;65(6):907‑11.**

**[94].  Laidemitt MR, Brant SV, Mutuku MW, Mkoji GM, Loker ES. The diverse echinostomes from East Africa: With a focus on species that use Biomphalaria and Bulinus as intermediate hosts. Acta Trop. 1 mai 2019;193:38‑49.** 
