# Supplementary material for: MALDI-TOF mass spectrometry for the identification of freshwater snails from Senegal, including intermediate hosts of schistosomes
Source: PLoS Negl Trop Dis. 2021 Sep 13;15(9):e0009725. doi: 10.1371/journal.pntd.0009725 (PMC8489727; doi:10.1371/journal.pntd.0009725)
Supplement: Attachment — Submitted filename: Hamlili_Author responses_August.docx [file pntd.0009725.s011.docx]

>Ms. Ref. No.:  PNTD-D-20-02023R1

>Title: MALDI-TOF mass spectrometry for the identification of freshwater snails from Senegal, including intermediate hosts of schistosomes.

>Running title: Identification of freshwater snails using MALDI-TOF MS

>PLOS NTD

Reviewer #1

1- **The authors should deposit all the MALDI-MS data in a public repository such as ProteomeXchange, Massive and others to make available for the entire research community. This reviewer considers extremely important this point since the raw data should be publicly shared with the research community.**

We have deposited our Raw MS spectra from frozen, and ethanol-stored snail specimens included in the reference MS spectra database in a public repository in an attempt to be shared with the entire research community as suggested by the reviewer. The MS spectra are available and can be downloaded with the DOI number: <https://doi.org/10.35088/f605-3922>. We have added in the data availability section the DOI of MS spectra of the raw data as follows: **Line 627-628 “****snail MALDI-TOF MS database is publicly accessible and can be downloaded with the following DOI number:** [**https://doi.org/10.35088/f605-3922**](https://doi.org/10.35088/f605-3922)**.**

**2- Please state in the legend of Figure 3 the protocol that was chosen to build the reference database and perform the analysis.**

Thank you for this remark. The protocol used for MS database creation and analysis was added in the legend of Figure 3 as suggested.

**Line 1001-1002:** **The protocol F was selected to build the MS reference database and perform the analysis.**

--------------------

Reviewer #2:

**1- Line 38: log-score instead of log score**

Thank you for this remark. The correction has been made as suggested.

**Line 38: log-score.**

**2- Line 76: the sentence «…, whereas  S. mekongi, S. guineensis  and S. intercalatum have a lower global prevalence » is not clear as these species have a restricted geographic distribution (South-East asia   for S. mekongi, Cameroon, Equatorial Guinea, Gabon, Nigeria, and Sao Tomé for S. guineensis). I am not sure that it is necessary to mention these species in the manuscript.**

As suggested by the reviewer, the following sentence: **whereas S. mekongi, S. guineensis  and S. intercalatum have a lower global prevalence**, has been removed from line 76.

**2- Line 274-276: “****The visual comparison of MS profiles using an unsupervised statistical test (PCA, ClinProtools software) revealed that the dots corresponding to MS spectra from H2,  FH, F are separated from those of H1, H3 (Fig 3B).” instead of  “The visual comparison of MS profiles using an unsupervised statistical test (PCA, ClinProtools software) revealed a separation of the dots corresponding to MS spectra from H2,  FH, F and H1, H3 (Fig 3B).”**

The text has been revised as suggested. **Line 275-277 “The visual comparison of MS profiles using an unsupervised statistical test (PCA, ClinProtools software) revealed that the dots corresponding to MS spectra from H2, FH, F are separated from those of H1, H3”**

**3-    Line 289:** **127 of the 186 frozen snail instead of “127 of the 186 snail ».**

The reviewer is right. The suggested correction has been made: **Line 290-291 “127 of the 186**

**frozen snail”**

**4-    Line 399 : « …as Stephan et al. were the first… » or « …as the paper by Stephan et al. was the first… » instead of « …as Stephan et al. was the first… »**

We thank the reviewer for this remark. The error has been fixed. We kept the first suggestion. **Line 403: as Stephan *et al.* were the first**.

**5-    Line 514 : « it could be used to circumvent molecular limitations. ». It is not clear for me which molecular limitation are circumvent. Are these the limitations related to the price and the time required for molecular biology?**

The reviewer raised a good question. Yes, it is related to the time required and the price of molecular biology. Since the MALDI-TOF MS allowed the rapid identification of snail species, we stated that it could be used to overcome those limitations. For instance, sequencing techniques are time-consuming and laborious compared to the MALDI-TOF MS technique which is rapid and simple. Also, the molecular techniques require the high-cost of reagents, the availability and the of target sequences in the GenBank as the latter is still incomplete for some species or can be inaccurate for other species (wrong initial identification). As well, the correct molecular identification is conditioned by the choice of the right target gene and good quality reference Genbank sequences. We raised in our paper the reliability of molecular tools for the identification of the genus *Bellamya* as we could not confirm the species *Be. unicolor* molecularly. Precisely, the genes used were not that discriminant for the *Bellamya* genus. For more clarification, we reformulated the paragraph as follows:

**Line 514-517 “****we showed that MALDI-TOF MS is a reliable tool for the identification of different frozen snail species.** **It could be used to circumvent molecular limitations such as the long time required for sample preparation, high-cost reagents, and even the lack of reference data”**

**6-    Line 556: organic preservation solution instead of « organic buffer » as ethanol has no buffering effect.**

The reviewer is right. The correction has been made as suggested. **Line 559-560:** **organic preservation solution**

**7-    Line 586: “…efficient to discriminate two cryptic species that are hard to be distinguished morphologically… » If I understand correctly the specimens used for the blind test came from the second field collection and were not identified by molecular biology. It is therefore not possible to be sure if MALDI-TOF is able to discriminate morphologically close species such as B. senegalensis and B. forskalii.**

Thank you for pointing out this comment. We did not sequence all the specimens that came from the second field collection. However, the specimens of which the MS spectra were implemented in the MS database were confirmed molecularly. For the *Bu. senegalensis* and *Bu. forskalii*, the two species are morphologically close, and it is hard to be distinguished. In our study, we obtained the MS spectra from one specimen of *Bu. senegalensis* (confirmed molecularly) and some specimens of *Bu. forskalii* (confirmed molecularly). In the MS dendrogram, *Bu. senegalensis* clustered differently from *Bu. forskalii*. Even if we obtained MS spectra from one specimen of *Bu. senegalensis*, it is difficult to draw a conclusion on the ability of MALDI-TOF MS to distinguish these two species. On the other hand, previous studies stated that this tool could distinguish close species. For instance, the robustness of MALDI-TOF MS was reported to accurately identify taxonomically closely related mosquitoes: *Anopheles gambiae* complex (Müller *et al.,* 2013). Another study also reported that this tool was reliable for identifying closely related species belonging to the *Ixodes* genus that are hardly distinguishable (Boyer *et al.,* 2019). Therefore, we suppose that the MALDI-TOF MS might be used for the closely related species belonging to the genus *Bulinus* such as *Bu. senegalensis* and *Bu. forskalii*. We reformulated the sentence as follows: **Line 588-590**“**This high-throughput approach is rapid, low-cost, and could be used for closely related species belonging to the genus *Bulinus*, such as *Bu. senegalensis* and *Bu. forskalii* which are hardly differentiated morphologically**”

**References**

-Boyer PH, Almeras L, Plantard O, Grillon A, Talagrand-Reboul É, McCoy K, Jaulhac B, Boulanger N. Identification of closely related Ixodes species by protein profiling with MALDI-TOF mass spectrometry. PLoS One. 2019 Oct 17;14(10):e0223735. doi: 10.1371/journal.pone.0223735. PMID: 31622384; PMCID: PMC6797106.

-Müller P, Pflüger V, Wittwer M, Ziegler D, Chandre F, Simard F, et al. (2013) Identification of Cryptic Anopheles Mosquito Species by Molecular Protein Profiling. PLoS ONE 8(2): e57486. https://doi.org/10.1371/journal.pone.0057486 we claimed that the MALDI-TOF MS might be useful to differentiate these species

**8-    Line 1012 – Figure 5: Panel D, mentioned in legend of figure 5, is missing in the file Figure5.tiff**

We would like to thank the reviewer for this important comment. The panel D was added to the figure 5.

**9-    Figure 2: In my opinion, adding this figure helped clarify the workflow. Perhaps, if that does not overload the figure, it would be interesting to mention, in boxes B and C, the species respectively used for the creation of the base and for the blind test. Or alternatively, refer to the tables concerned.**
Thank you for this important comment. We chose the second option as suggested by the reviewer. We referred the table 2 and 3 in the flowchart to clarify more the workflow.
